# Synthesis and Performance Evaluation of High-Temperature-Resistant Extreme-Pressure Lubricants for a Water-Based Drilling Fluid Gel System

**DOI:** 10.3390/gels10080505

**Published:** 2024-08-01

**Authors:** Shengming Huang, Tengfei Dong, Guancheng Jiang, Jun Yang, Xukun Yang, Quande Wang

**Affiliations:** 1 College of Petroleum Engineering, Ministry of Education (MOE) Key Laboratory of Petroleum Engineering, China University of Petroleum (Beijing), Beijing 102249, China; smhuang2015@163.com (S.H.); 18801281480@163.com (J.Y.); yangxukunbj@126.com (X.Y.); 15664628289@163.com (Q.W.); 2 State Key Laboratory of Petroleum Resources and Prospecting, China University of Petroleum (Beijing), Beijing 102249, China; 3 National Engineering Research Center of Oil & Gas Drilling and Completion Technology, Beijing 102206, China; 4 College of Science, China University of Petroleum (Beijing), Beijing 102249, China

**Keywords:** extreme-pressure lubricants, high-temperature resistance, lubrication retention performance, lubrication mechanism, water-based drilling fluid gel system

## Abstract

Addressing the high friction and torque challenges encountered in drilling processes for high-displacement wells, horizontal wells, and directional wells, we successfully synthesized OAG, a high-temperature and high-salinity drilling fluid lubricant, using materials such as oleic acid and glycerol. OAG was characterized through Fourier-transform infrared (FTIR) spectroscopy and thermogravimetric analysis (TGA). The research findings demonstrate the excellent lubricating performance of OAG under high-temperature and high-salinity conditions. After adding 1.0% OAG to a 4% freshwater-based slurry, the adhesion coefficient of the mud cake decreased to 0.0437, and at a dosage of 1.5%, the lubrication coefficient was 0.032, resulting in a reduction rate of 94.1% in the lubrication coefficient. After heating at 200 °C for 16 h, the reduction rate of the lubrication coefficient reached 93.6%. Even under 35% NaCl conditions, the reduction rate of the lubrication coefficient remained at 80.3%, indicating excellent lubrication retention performance. The lubricant OAG exhibits good compatibility with high-density drilling fluid gel systems, maintaining their rheological properties after heating at 200 °C and reducing filtration loss. The lubrication mechanism analysis indicates that OAG can effectively adsorb onto the surface of N80 steel sheets. The contact angle of the steel sheets increased from 41.9° to 83.3° before and after hot rolling, indicating a significant enhancement in hydrophobicity. This enhancement is primarily attributed to the formation of an extreme-pressure lubricating film through chemical reactions of OAG on the metal surface. Consequently, this film markedly reduces the friction between the drilling tools and the wellbore rocks, thereby enhancing lubrication performance and providing valuable guidance for constructing high-density water-based drilling fluid gel systems.

## 1. Introduction

As the depth of drilling formations increases during the exploration of oil and gas resources, the complexity of encountered formations grows. This complexity is com-pounded by the increasing prevalence of deep wells, high-displacement wells, horizontal wells, and directional wells. These complex well structures present new challenges for safe and efficient drilling, including issues such as high friction, high torque, sticking, and bit balling [1,2,3,4]. High friction and torque in high-displacement and horizontal drilling are critical factors limiting their lateral extension. Friction between the drilling tools and drilling fluid, friction between the drilling tools and the wellbore (mud cake or formation rock), and the cutting and crushing of rock by the drill bit are the main sources of torque and friction during the drilling process [5,6,7]. High torque and friction pose numerous challenges to drilling operations, including underground accidents such as stuck pipes, reduced mechanical drilling speeds, and increased drilling costs [8,9,10,11]. Addressing these challenges requires the development of efficient lubricants capable of adapting to the complex conditions of high temperature, high salinity, and high density in formations [12].

The primary function of drilling fluid lubricants is to reduce friction between the drilling tools and the wellbore rocks, thereby minimizing losses due to friction, extending the service life of drilling tools, and effectively preventing underground accidents such as differentially stuck pipes. The adhesion coefficient of the mud cake and the lubrication coefficient of the drilling fluid are the two most commonly used indicators to evaluate the lubricating performance of drilling fluids [13,14]. According to the principles of tribology, dry friction and fluid lubrication represent two extremes of lubrication states, with dry friction having the worst lubricating properties and fluid lubrication exhibiting the best performance, while drilling fluid lubrication falls between the two. Well-formulated drilling fluids with good lubricating properties can reduce tool wear, prevent sticking, decrease tripping time, enhance mechanical drilling speed, and lower operational costs [15,16,17,18,19]. Drilling fluid lubricants can be mainly classified into solid lubricants and liquid lubricants based on their phase states [20,21]. Solid lubricants mainly include plastic balls, graphite, glass beads, and others. They function between the friction surfaces to transform sliding friction into rolling friction, significantly reducing drilling torque and friction. However, during usage, solid lubricants tend to have larger particles, making them prone to separation by solid-phase control equipment in drilling fluids. Moreover, they are susceptible to extrusion or shear damage, leading to loss of lubricating effectiveness [22,23]. Currently, there is an extensive research and application of liquid lubricants by scholars. Liquid lubricants mainly include alcohol ether, ester, amide, and vegetable oil types, with alcohol ether and polyol ester liquid lubricants predominantly used in big extended wells and horizontal wells. As environmental requirements increase, high-performance yet heavily polluting liquid lubricants such as asphalt-based, diesel-based, and mineral oil-based ones are gradually disappearing from the market. Instead, environmentally friendly lubricants, extreme-pressure lubricants, and nanolubricants are becoming the focus of research both domestically and internationally [24,25,26,27]. Ester-based lubricants exhibit excellent lubricating performance and are readily biodegradable in natural environ-ments, making them environmentally friendly lubricants. They can be used in both high- and low-temperature conditions and offer lubrication comparable to oil-based drilling fluids [28,29,30]. In the 1990s, ester lubricants were tested as additives in water-based drilling fluids in the Norwegian Sea, achieving significant success. Subsequently, C12-C14 ester-based drilling fluid systems have been widely adopted globally [31,32]. Argillier et al. [33] showed that ester-based lubricants help reduce torque, resistance, and increase the drilling speed in water-based drilling fluids during directional drilling. Additionally, Okaro et al. [34] investigated the suitability of thermally cracked oil from waste tires as an additive in water-based drilling fluids in recent years. This additive demonstrates excellent antifriction properties. Perumalsamy et al. [26] studied the influence of two additives, PC60 (the product of the reaction of glycerol + tall oil fatty acid) and graphene particles, on the rheological and lubricating properties of a water-based drilling fluid gel system at different temperatures. The research indicates that the viscosity of prepared samples decreases with an increasing temperature. Samples containing 3% PC-60 exhibited better rheological and lubricating properties before hot rolling at 30 °C and 60 °C. Ma et al. [35] modified by sulfuration and esterification a by-product of vegetable oil to synthesize an environmentally friendly composite water-based drilling fluid lubricant. This lubricant demonstrates excellent performance under conditions of 180 °C heat resistance and high salinity. However, under extremely high-temperature, salinity, and density conditions, most lubricants exhibit insufficient performance. Therefore, there is a need to develop an efficient drilling fluid lubricant suitable for deep formation drilling. Wang et al. [36] reported the use of an organic borate ester SOP as an environmentally friendly drilling fluid lubricant, and the evaluation results showed that the addition of 1% lubricant SOP to the freshwater-based slurry reduced the lubrication coefficient from 0.631 to 0.046, and the lubrication effect was obvious. Geng et al. [37] prepared a mixed lubricant of graphene and trioleic acid for water-based drilling fluids, and the adhesion coefficient of the drilling fluids was reduced to as low as 0.055 at 240 °C, with a reduction rate of more than 70%. In recent years, scholars at home and abroad have conducted extensive research on lubricants for water-based drilling fluids and achieved good results. Esters of oleic acid have received great attention from industry professionals due to their excellent lubricating properties, thermal stability, and low toxicity.

In this study, we successfully synthesized a high-temperature-resistant lubricant, OAG, using oleic acid and glycerol as the raw materials. OAG was characterized using FTIR and TGA. We evaluated the lubricating performance of OAG as a water-based drilling fluid lubricant in both base mud and high-density drilling fluid gel systems. Additionally, we analyzed the lubrication mechanism of OAG. It is anticipated that our findings will provide valuable insights into drilling operations for complex well types such as deep wells, high-displacement wells, and horizontal wells, as well as the construction of high-density water-based drilling fluid gel systems.

## 2. Results and Discussion

### 2.1. Characterization of the OAG

#### 2.1.1. Fourier-Transform Infrared (FTIR) Spectroscopy

**Figure 1:**
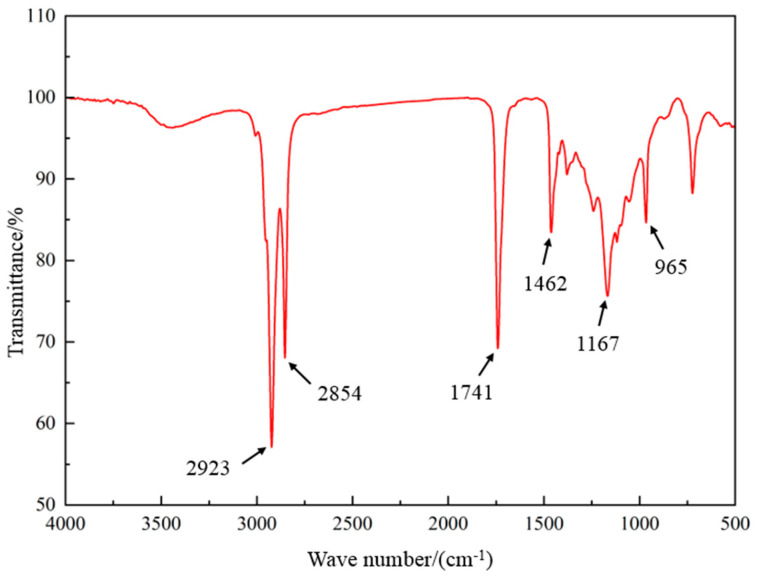
FTIR spectra of OAG.

According to the analysis method of infrared spectroscopy in Section 4.3, the synthesized lubricant OAG was subjected to FTIR analysis using the KBr pellet method. The scan results are shown in Figure 1. Among them, the peak at 2854 cm^−1^ corresponds to the symmetric stretching vibration of methylene (-CH_2_) groups, the peak at 1741 cm^−1^ corresponds to the stretching vibration of ester carbonyl (C=O) groups, the peak at 1462 cm^−1^ corresponds to the asymmetric bending vibration of methyl (-CH_3_) groups, the peak at 1167 cm^−1^ corresponds to the stretching vibration of the C-O-C group in esters, and the peak at 965 cm^−1^ corresponds to the out-of-plane bending vibration of carbon–carbon double bonds (C=CH_2_). In conclusion, the presence of ester carbonyl and C-O-C structures adequately demonstrates the esterification reaction between oleic acid and glycerol, resulting in the formation of the target product OAG.

#### 2.1.2. Thermogravimetric Analysis (TGA)

**Figure 2:**
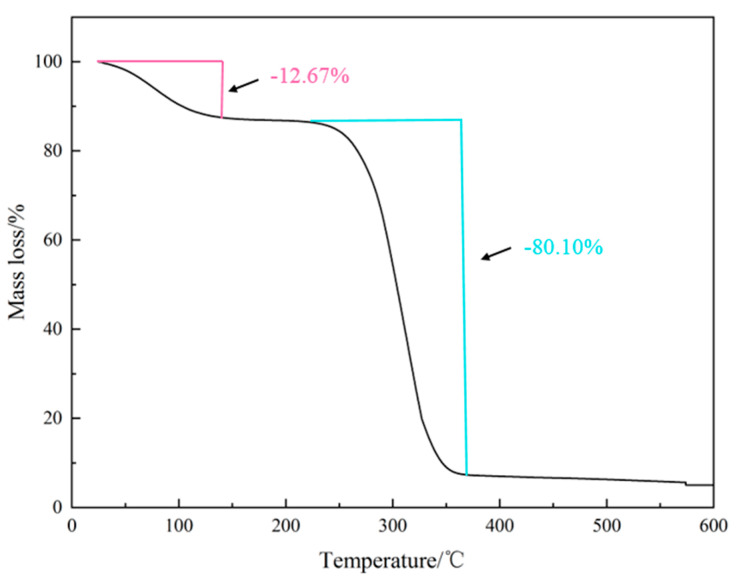
Thermal gravimetric curves of OAG.

Following the thermal analysis method described in Section 4.3, an appropriate amount of sample was weighed, and TGA was performed. The results are depicted in Figure 2. As the temperature increases, the lubricant OAG exhibits a downward trend in mass loss. The TGA curve can be divided into two stages. In the first stage, before 145 °C, the mass loss of OAG is 12.67%. This initial mass loss is primarily attributed to the evaporation of moisture from both intra- and intermolecular sources. In the second stage, as the temperature rises to 375 °C, the mass loss of OAG reaches 80.10%. This stage experiences the most severe mass loss, mainly due to the degradation of long-chain fatty acids and ester groups in the product. Therefore, from the TGA curve, it can be observed that OAG exhibits good thermal stability up to 230 °C, beyond which the mass loss begins to decrease continuously.

### 2.2. Performance Evaluation of OAG

#### 2.2.1. Lubricating Performance Test in Freshwater-Based Slurry

**Figure 3:**
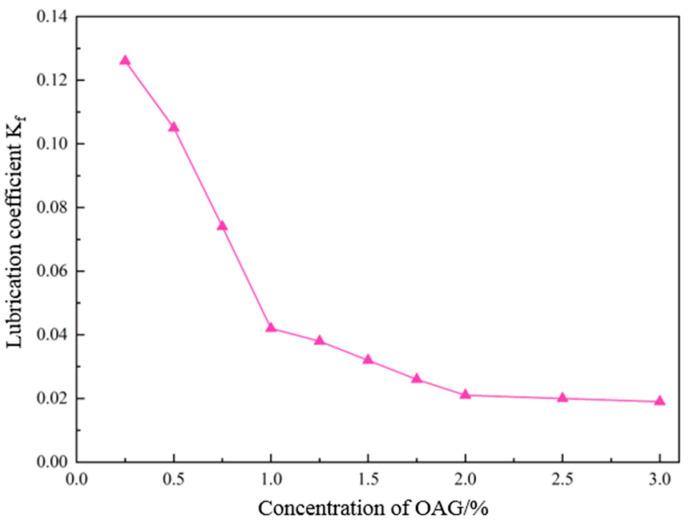
Effect of different concentrations of OAG on the lubrication coefficient of freshwater-based slurry.

Incorporating different mass concentrations of lubricant OAG into 4.0% freshwater-based slurry, the effect of lubricant OAG on the lubricating performance of the base slurry was tested using an EP-C extreme-pressure lubrication tester. The test results are presented in Figure 3. Before adding OAG, the lubrication coefficient of the 4.0% freshwater-based slurry was 0.541. Upon adding 1.0% OAG, the lubrication coefficient of the base slurry decreased to 0.042, with a reduction rate of 92.2%. With the addition of 1.5% OAG, the lubrication coefficient further decreased to 0.032, with a reduction rate of 94.1%. Subsequently, as the concentration of OAG was increased, the reduction in the lubrication coefficient of the base slurry diminished. When the concentration of OAG was increased from 2.0% to 3.0%, the lubrication coefficient of the base slurry remained essentially unchanged. In conclusion, as the concentration of lubricant OAG increases, the lubrication coefficient of the base slurry gradually decreases. When a certain concentration is reached, the lubrication coefficient of the base slurry remains relatively stable. Considering both the economic cost and the efficacy of lubricant OAG, the recommended dosage of OAG is around 1.0–1.5%.

**Figure 4:**
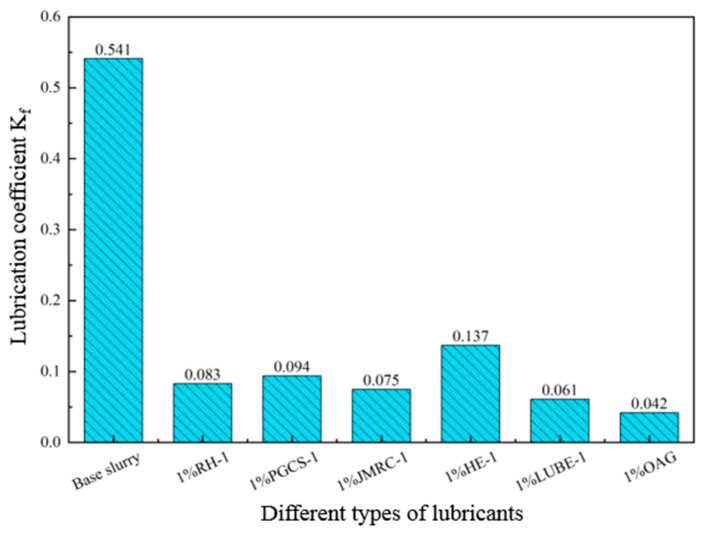
Effect of different types of lubricants on the lubrication coefficient of freshwater-based slurry.

**Figure 5:**
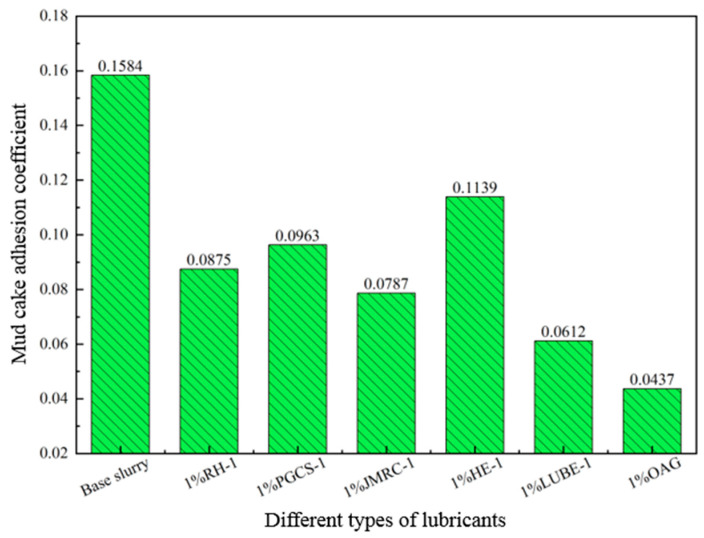
Effect of different types of lubricants on the adhesion coefficient of mud cakes in freshwater-based slurry.

In the 4.0% freshwater-based slurry, different types of lubricants were added to evaluate and compare the lubrication performance of OAG with other types of lubricants. The test results, as shown in Figure 4, indicate that when 1.0% of RH-1, PGCS-1, JMRC-1, HE-1, and LUBE-1 were added, the lubrication coefficient of the base slurry decreased from 0.541 to 0.083, 0.094, 0.075, 0.137, and 0.061, respectively. However, when 1.0% of OAG was added, the lubrication coefficient of the base slurry decreased to 0.042. It can be observed that under the same concentration, OAG exhibits excellent lubrication performance. Simultaneously, the adhesion coefficient of mud cakes in 4.0% freshwater-based slurry was tested for different types of lubricants, and the test results are shown in Figure 5. The adhesion coefficient of the base slurry mud cake was 0.1584. After adding other lubricants, the adhesion coefficient of the mud cake significantly decreased. With the addition of 1.0% RH-1, PGCS-1, JMRC-1, HE-1, and LUBE-1, the adhesion coefficient of the mud cake decreased from 0.1584 to 0.0875, 0.0963, 0.0787, 0.1139, and 0.0612, respectively. However, after adding 1.0% OAG, the adhesion coefficient of the mud cake decreased to 0.0437, indicating that OAG exhibited a lower mud cake adhesion coefficient. In summary, the lubricating effect of OAG surpasses that of other lubricants, primarily because OAG can form a layer of extreme-pressure lubricating film on the metal surface, significantly reducing the sliding resistance between the drilling tools.

#### 2.2.2. Temperature-Resistance Performance

**Figure 6:**
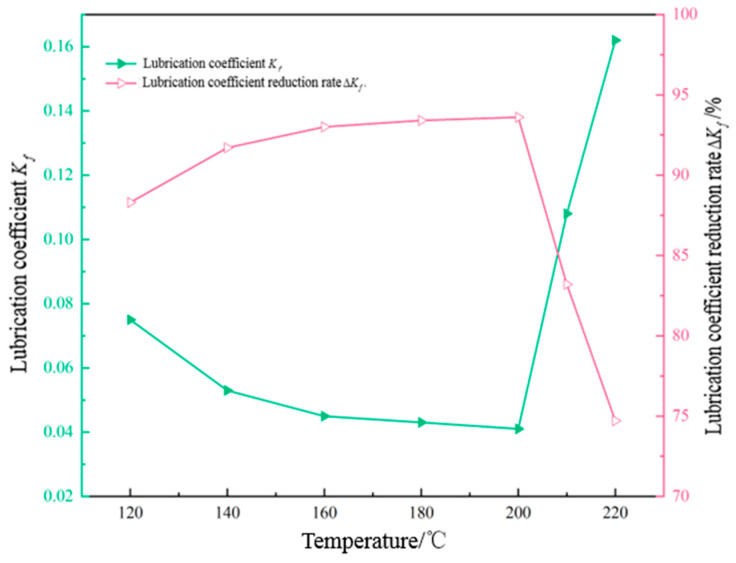
Lubrication performance of OAG at different temperatures.

**Figure 7:**
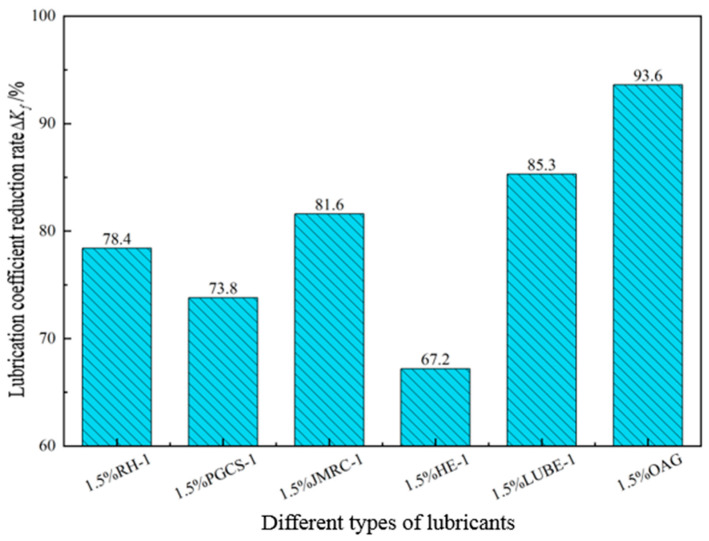
Lubrication performance of different lubricants after hot rolling at 200 °C.

A certain amount of 4% freshwater-based slurry was taken and an EP-C extreme-pressure lubricant tester was used to evaluate the lubricating performance of the base slurry with 1.5% OAG added after hot rolling at different temperatures for 16 h, to assess the temperature resistance of the lubricant OAG. Additionally, the lubrication coefficient reduction rates of different types of lubricants after hot rolling at 200 °C were tested, as shown in Figure 6 and Figure 7. From Figure 6, it can be observed that when the hot rolling temperature is below 200 °C, the lubrication coefficient reduction rate of the base slurry with 1.5% OAG increases from 88.3% to 93.6% with increasing temperature. However, when the hot rolling temperature exceeds 200 °C, the lubrication coefficient reduction rate starts to decrease. As shown in Figure 7, it can be observed that after adding 1.5% of RH-1, PGCS-1, JMRC-1, HE-1, and LUBE-1 following hot rolling at 200 °C, the lubrication coefficient reduction rates of the base slurry are 78.4%, 73.8%, 81.6%, 67.2%, and 85.3%, respectively. However, when adding the same concentration of OAG, the reduction rate of the lubrication coefficient of the base slurry is significantly higher than that of other types of lubricants. This indicates that the temperature-resistance performance of OAG is superior to other lubricants. Even when the hot rolling temperature increases to 210 °C, the reduction rate of the lubrication coefficient of the base slurry still maintains at 83.2%, demonstrating the excellent temperature-resistance capability of OAG.

#### 2.2.3. Salt-Resistance Performance

**Figure 8:**
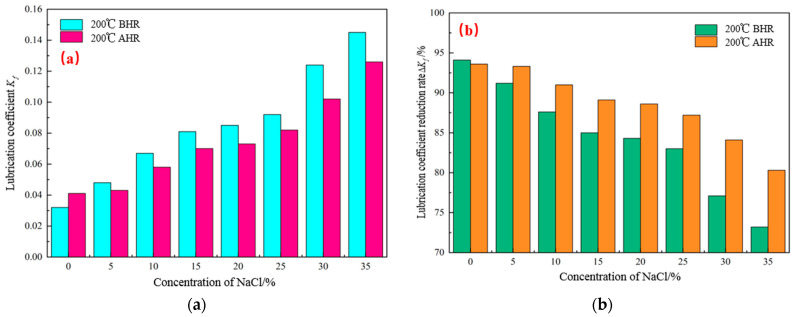
Salt-resistance testing of lubricant OAG (BHR: before hot rolling; AHR: after hot rolling). (**a**) Lubrication coefficient of OAG at different NaCl concentrations, (**b**) lubrication coefficient reduction rate of OAG at different NaCl concentrations.

**Figure 9:**
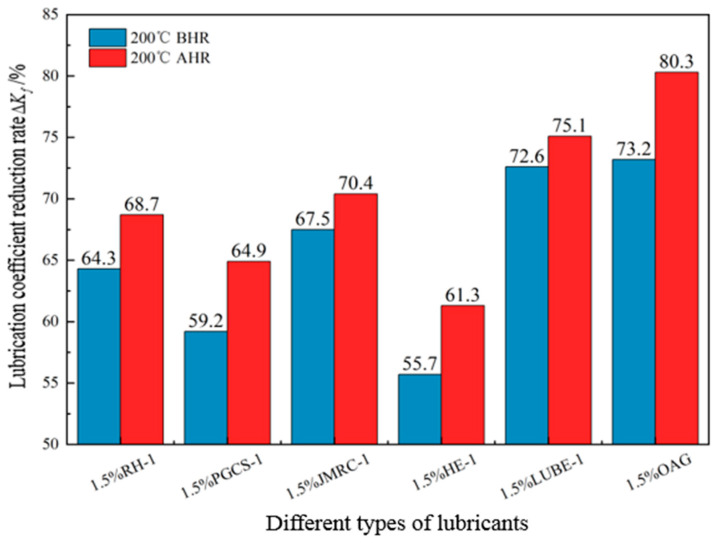
Reduction rate of the lubrication coefficient of different lubricants under 35% NaCl conditions.

A certain amount of 4% freshwater-based slurry was taken, and the lubrication performance of the base slurry with 1.5% OAG added was tested using the EP-C extreme-pressure lubrication tester after hot rolling at 200 °C for 16 h under different NaCl concentrations, to investigate the salt-resistance performance of the lubricant OAG. The test results are shown in Figure 8. As shown in Figure 8, it can be observed that with the increase in NaCl concentration, the lubrication coefficient of the base slurry before and after hot rolling also increases. When the NaCl concentration increases from 0% to 35%, the lubrication coefficient after hot rolling increases from 0.041 to 0.126. Meanwhile, the reduction rate of the lubrication coefficient of the base slurry decreases from 93.6% to 80.3%. However, the overall reduction rate of the lubrication coefficient after hot rolling still remains above 80%. Additionally, the lubrication coefficient reduction rates of different lubricants after hot rolling at 200 °C for 16 h under 35% NaCl conditions were also tested, and the results are presented in Figure 9. As depicted in Figure 9, it is evident that in base slurry containing 1.5% RH-1, PGCS-1, JMRC-1, HE-1, and LUBE-1, the reduction rates of the lubrication coefficient after hot rolling with 35% NaCl are 68.7%, 64.9%, 70.4%, 61.3%, and 75.1%, respectively. However, in the base slurry with OAG lubricant, the reduction rate of the lubrication coefficient is 80.3%. This indicates that the salt-resistance performance of the OAG lubricant is superior to that of the tested RH-1, PGCS-1, JMRC-1, HE-1, and LUBE-1 lubricants. In summary, the OAG lubricant can maintain good lubrication performance under high salt conditions, demonstrating its excellent resistance to temperature and salt.

#### 2.2.4. Lubrication Retention Performance

A certain amount of 4% fresh water-based slurry was taken and the EP-C extreme-pressure lubrication instrument was used to test the lubrication coefficient and the heating of the slider at different extreme-pressure friction times before and after adding 1.5% OAG to the base slurry, to evaluate the lubrication effectiveness of the OAG lubricant. The test results are shown in Table 1. When no OAG lubricant is added, the lubrication coefficient of the base slurry continuously increases with the increase in extreme-pressure friction time, and the slider also heats up. However, after adding 1.5% of the OAG lubricant, the lubrication coefficient of the base slurry decreases rapidly and slightly decreases with the increase in extreme-pressure friction time, and the slider does not heat up. When the extreme-pressure time is 30 min, the lubrication coefficient of the 4% base slurry is 0.6074, while after adding 1.5% OAG lubricant, the lubrication coefficient decreases to 0.0328. In summary, the OAG lubricant forms a high-strength, extreme-pressure lubricating film on the metal surface, enhancing the lubrication effect of the base slurry, thereby demonstrating excellent lubrication effectiveness.

#### 2.2.5. Compatibility of OAG with a Drilling Fluid Gel System

The effect of OAG lubricant dosage on the performance of both 4% freshwater-based slurry and high-temperature, high-density drilling fluid gel systems was investigated. The hot rolling conditions were set at 200 °C for 16 h. The test results are shown in Table 2. The drilling fluid formulation consisted of 400 mL clean water + 4% bentonite + 0.35% Na_2_CO_3_ + 0.5% NaOH + 0.1% FA-367 + 1.5% DSP-1 + 2.5% SPNH + 1.5% SMP-1 + 1.5% NP-1 + 35% NaCl + barite, with a density of 2.1 g/cm^3^.

As depicted in Table 2, it can be observed that in the 4% freshwater-based slurry, the effect of OAG on the rheological properties of the slurry was studied. After the addition of OAG and hot rolling, there was a slight increase in the viscosity of the slurry, although not significantly. Additionally, as the concentration of OAG increased, the fluid loss of the slurry decreased. This indicates that the lubricant OAG does not adversely affect the rheological properties of the slurry and can even improve the filtration wall–building properties of the slurry. In high-density drilling fluids, with the addition of lubricant OAG, there is a slight increase in the viscosity of the drilling fluid, with overall performance changes being minimal. However, the addition of OAG results in a reduction in fluid loss, indicating that OAG does not affect the rheological properties of high-density drilling fluids and can reduce their fluid loss. Additionally, the lubrication coefficient significantly decreases after adding OAG, with a reduction rate of 69.0% in the lubrication coefficient after hot rolling when OAG concentration is 1.5%. This suggests that lubricant OAG has good compatibility with high-density drilling fluids and can improve the lubrication performance of a drilling fluid gel system.

### 2.3. Lubrication Mechanism Analysis

#### 2.3.1. Adsorption Performance

**Figure 10:**
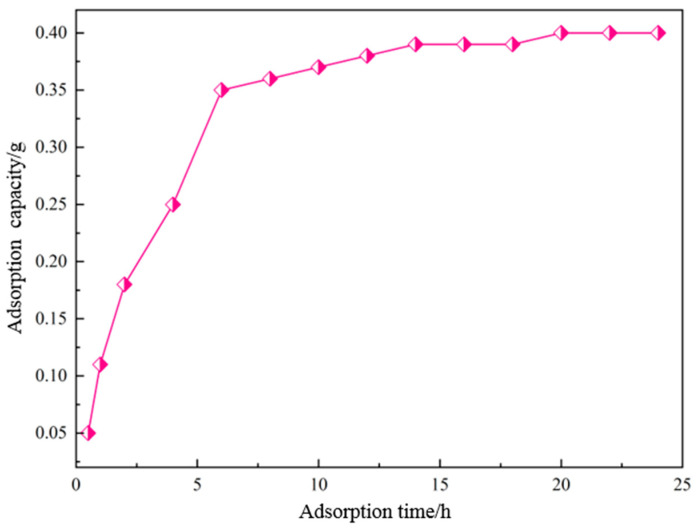
Variation curve of lubricant OAG adsorption amount with adsorption time.

**Figure 11:**
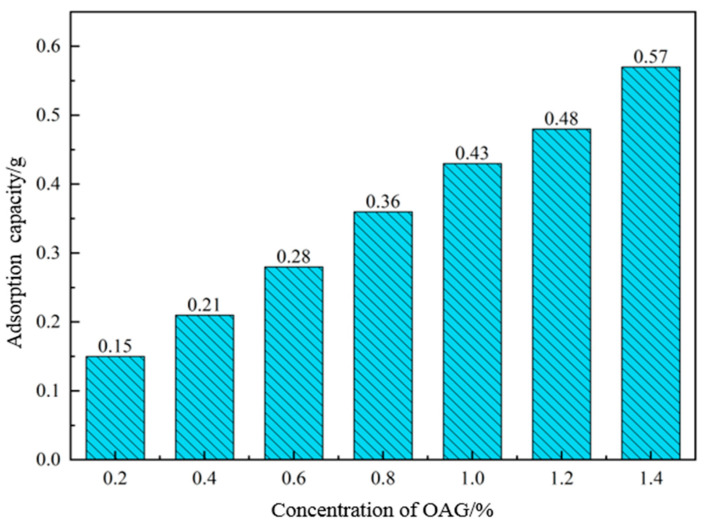
Variation curve of adsorption with lubricant OAG concentration.

The adsorption capacity can reflect the interaction between the lubricant and the drill string. The greater the adsorption of the lubricant on the drill string, the better the lubricating effect of the lubricant. Therefore, we chose N80 steel sheets immersed in the lubricant solution to simulate the adsorption of the lubricant on the drill string. The adsorption of the lubricant on the steel sheets was determined using a UV-visible spectrophotometer, with an adsorption time of 24 h. The test results are shown in Figure 10 and Figure 11. From Figure 10, it can be observed that in a 1.5% OAG lubricant solution, the adsorption of OAG on the steel sheet reaches 0.35 g after 6 h of adsorption. Subsequently, the adsorption rate increases slowly, and after 24 h, the adsorption amount reaches 0.4 g. As depicted in Figure 11, it can be observed that as the concentration of the lubricant OAG increases from 0.2% to 1.4%, the adsorption of OAG on the steel sheet increases from 0.15 g to 0.57 g. This indicates that a higher concentration of OAG leads to greater adsorption on the steel sheet, thereby enhancing its lubricating effect. In summary, lubricant OAG can effectively adsorb on the surface of the steel sheet, forming an extreme-pressure lubricating film, thereby reducing the friction between the drilling tools and the wellbore, and significantly reducing the lubrication coefficient between the drilling fluid and the drilling tools.

#### 2.3.2. Wetting Performance

**Figure 12:**
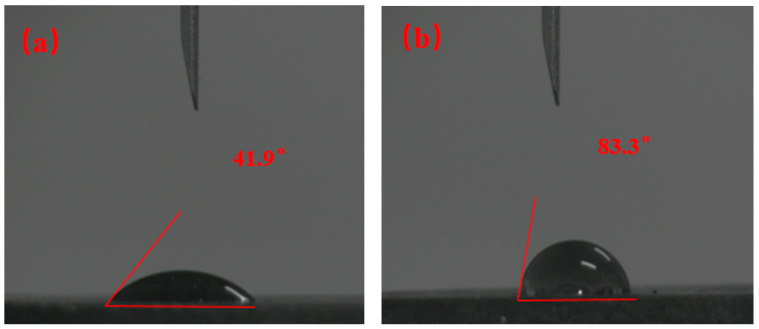
Contact angles of N80 steel sheets in base slurry containing OAG: (**a**) BHR, (**b**) AHR.

The N80 steel sheet was subjected to hot rolling in a freshwater-based slurry containing lubricant OAG. After hot rolling, it was removed and dried. The contact angle of the N80 steel sheet surface before and after hot rolling was tested using deionized water to assess the wetting performance of the lubricant OAG on the metal surface. The test results are shown in Figure 12. The contact angle of the steel sheet increased from 41.9° to 83.3° before and after hot rolling, indicating a significant enhancement in hydrophobicity. This suggests that the lubricant OAG formed a hydrophobic adsorption film on the surface of the N80 steel sheet, gradually changing the wettability of the steel sheet surface from hydrophilic to hydrophobic. This is mainly attributed to the molecular structure of the lubricant OAG, which contains hydroxyl and ester groups, exhibiting strong adsorption capacity to form an adsorption film on the metal surface. The hydrophobic long-chain structure can create a lubricating layer, reducing the friction between the drill string and the wellbore formation, thereby providing lubrication.

#### 2.3.3. Mechanism of Action Analysis

**Figure 13:**
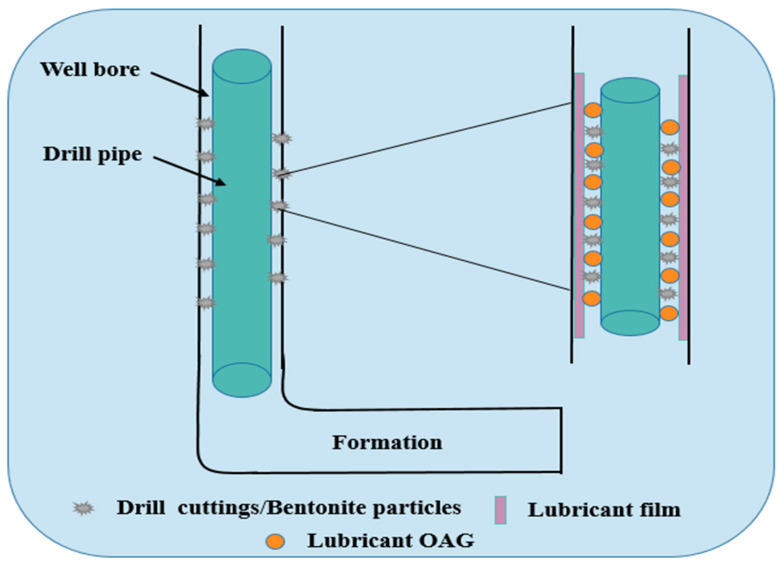
Mechanism of lubrication action of OAG in the formation.

The adsorption groups and extreme-pressure components present in lubricant OAG can undergo chemical reactions with metals under high-temperature conditions, forming a dense adsorption lubricating film on the surface of the drill string. This is because OAG can form metal coordination covalent bonds with metal ions (such as Fe^3+^) in metal oxides (e.g., Fe_2_O_3_), enhancing the toughness and wear resistance of the lubricating film [38]. Consequently, the contact between metal-to-metal and metal-to-rock transitions into contact between lubricating films, significantly reducing the friction between the drill string and the wellbore formation. Moreover, lubricant OAG forms a hydrophobic boundary layer on the metal surface, which helps reduce the dynamic friction generated when drilling fluid flows over the drill string surface. This effectively prevents prolonged frictional wear on the drill string surface, thereby achieving long-term lubrication. The mechanism of action of lubricant OAG in the formation is illustrated in Figure 13.

## 3. Conclusions

In this study, we successfully synthesized the high-temperature-resistant lubricant OAG using oleic acid and glycerol as the raw materials. The characterization of OAG was conducted through infrared spectroscopy and thermogravimetric analysis. We evaluated the lubrication performance of OAG as a water-based drilling fluid gel system lubricant in base slurry and high-density drilling fluid, and analyzed the lubrication mechanism of OAG. Experimental results show that the lubricant OAG exhibits high-temperature resistance up to 200 °C. After adding 1.0% OAG to a 4% freshwater-based slurry, the adhesion coefficient of the mud cake decreases to 0.0437. After hot rolling at 200 °C for 16 h, the reduction rate of the lubrication coefficient reaches 93.6%. Moreover, it exhibits good compatibility with high-density drilling fluid gel systems. At the same time, OAG can effectively adsorb on the metal surface to form a hydrophobic and high-strength lubricating adsorption film through a chemical reaction to form metal coordination covalent bonds, and change the wettability of the metal surface, effectively protect the surface of the drilling tools from adhesion and wear. As a type of high-efficient, high-temperature and salt-resistant lubricant, OAG can greatly reduce the friction and torque between the drilling tools and the wellbore rock, and thus serving a lubricating function and holding broad application prospects.

## 4. Materials and Methods

### 4.1. Materials and Instruments

#### 4.1.1. Materials

Oleic acid (CAS: 112-80-1), glycerol (ACS, 99.5%, CAS: 56-81-5), sodium chloride (NaCl, CAS: 7647-14-5), potassium chloride (KCl, CAS: 7447-40-7), magnesium chloride (MgCl2, CAS: 7786-30-3), sodium carbonate (Na_2_CO_3_, CAS: 497-19-8), sodium bicarbonate (NaHCO3, 99%, CAS: 144-55-8), and ethyl acetate (CAS: 141-78-6) were all purchased from the China National Pharmaceutical Group Corporation. p-Toluenesulfonic acid (99%, CAS: 104-15-4) and 1,4-dioxane (CAS: 123-91-1) were obtained from Shanghai Aladdin Chemical Reagent Co., Ltd. The sodium bentonite (Na-BT) for slurry preparation, lubricant RH-1, polyglycol compound PGCS-1, lubricant HE-1, and lubricant LUBE-1 were provided by the CNPC Research Institute of Engineering Technology. The high-efficiency and environmentally friendly lubricant JMRC-1 was provided by Suzhou Jinmuruncheng Technology Co., Ltd. (Suzhou, China) Deionized water was prepared in the laboratory, and all chemicals were dissolved in deionized water. All chemicals were used as received without further purification.

#### 4.1.2. Instruments

A variable-frequency, high-speed mixer (Tongchun Instruments, Qingdao, China) was employed for stirring drilling fluid systems. An SD-4 medium-pressure filtration tester (Chuangmeng Instruments, Qingdao, China) was utilized to measure the filtration loss of drilling fluid systems. A ZNN-D6 six-speed rotational viscometer (Haitongda Instruments, Qingdao, China) was used to test the viscosity of drilling fluid systems. An EP-C extreme-pressure lubrication tester (Senxin Machinery, Shanghai, China) was employed to measure the lubrication coefficient of drilling fluid systems. An NF-2 mud cake adhesion coefficient tester (Haitongda Instruments, Qingdao, China) was utilized to measure the mud cake adhesion coefficient of drilling fluid systems. A constant temperature vacuum drying oven (Jinghong Instruments, Shanghai, China) was used for drying and processing experimental samples. A high-temperature roller heating furnace (Tongchun Instruments, China) was employed for the thermal rolling treatment of drilling fluid systems. A UV-visible spectrophotometer (Jingcheng Instruments, Shanghai, China) was used for the analysis of the adsorption performance of drilling fluid lubricants. A JC2000D5M Contact Angle Tester (Zhongchen Instruments, Zhangzhou, China) was designed to measure the contact angle of N80 steel sheets before and after hot rolling in a freshwater substrate containing lubricant.

### 4.2. Preparation of the OAG

**Figure 14:**
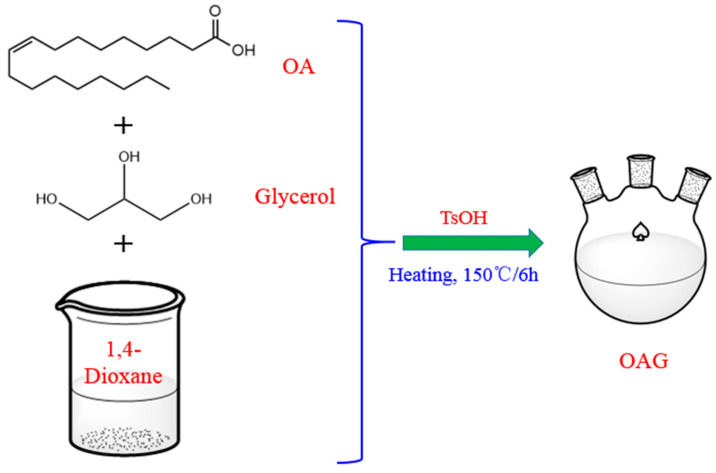
Schematic diagram of the OAG synthesis process.

**Figure 15:**
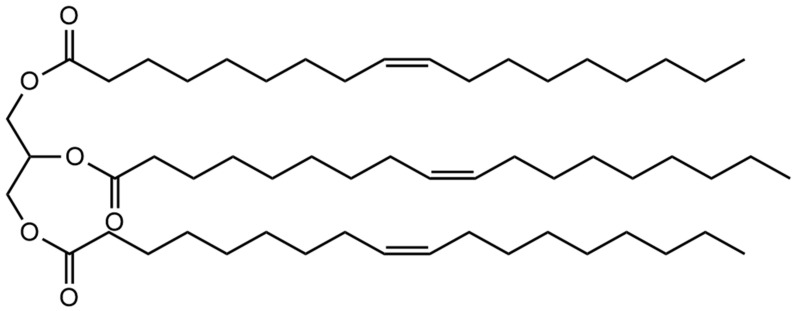
Structure of lubricant OAG.

A schematic diagram of the OAG synthesis process is shown in Figure 14. First, 0.2 mol of glycerol was poured into a three-necked flask equipped with a condenser and mixed with 0.1 mol of oleic acid. Then, both were added to the organic solvent 1,4-dioxane and stirred until completely dissolved. The reaction temperature was set at 150 °C and the reaction time was fixed at 6 h. Next, 0.01 mol of p-Toluenesulfonic acid was added as a catalyst and nitrogen gas was introduced before the reaction to remove oxygen. A separator containing anhydrous calcium chloride was used throughout the reaction process to eliminate the influence of water. After the completion of the reaction, the solvent was removed using a rotary evaporator. Subsequently, any unreacted small-molecule acids were eliminated using a NaHCO_3_ solution. Finally, water was removed via vacuum distillation, followed by drying at 80 °C to yield a brown viscous liquid, which is identified as the lubricant triolein glyceride (OAG) [39]. The structure of the OAG is shown in Figure 15.

### 4.3. Characterization of the OAG

The prepared water-based drilling fluid lubricant OAG was subjected to an FTIR spectroscopy instrument (Nicolet, Rhinelander, WI, USA) with 32 scans, within the range of 4000–500 cm^−1^ [40].

TGA of the prepared water-based drilling fluid lubricant OAG was conducted using an STA 449F5 instrument (NETZSCH, Waldkraiburg, Germany). The heating rate was set at 10 °C/min, and the testing temperature range was 25 to 600 °C under a nitrogen atmosphere [41].

### 4.4. Performance Evaluation Methods

#### 4.4.1. Preparation of Based Slurry

To prepare the 4% freshwater-based slurry, 400 mL of deionized water was measured using a graduated cylinder and 0.64 g of anhydrous NaCO_3_ and 16 g of sodium bentonite were added. The mixture was stirred thoroughly at 6000 rpm for 15–20 min, then sealed and incubated at room temperature for 24 h.

To prepare the saturated saltwater-based slurry, on the basis of preparing the 4% freshwater-based slurry, 400 mL was measured and 36% NaCl was added. The mixture was stirred thoroughly at 6000 rpm for 15–20 min, then sealed and incubated at room temperature for 24 h.

#### 4.4.2. Rheological Performance Test

According to the American Petroleum Institute (API) drilling fluid evaluation standards, the influence of OAG on water-based drilling fluid was studied through rheological and filtration tests. The rheological properties of the drilling fluid system were tested using a ZNN-D6 six-speed rotational viscometer. The drilling fluid was stirred at high speed for 15–20 min before and after thermal rolling at different temperatures. Then, the apparent viscosity (AV), plastic viscosity (PV), and yield point (YP) of the drilling fluid were determined, and the calculation formulas are as follows in Equations (1)–(3) [42]:

(1) $$AV\left(\mathrm{mPa}\cdot s\right)=0.5\times \varphi 600$$

(2) $$PV\left(\mathrm{mPa}\cdot s\right)=\varphi 600-\varphi 300$$

(3) $$YP\left(\mathrm{Pa}\right)=AV-PV$$

where φ600 and φ300 represent the readings at speeds of 600 r/min and 300 r/min, respectively. Additionally, Gel denotes the shear stress of the drilling fluid at 3 rpm after 10 s and 10 min of static condition.

#### 4.4.3. Lubricity Coefficient Test

The lubricity performance of the base slurry of water-based drilling fluid with different mass concentrations of lubricant OAG was tested using the EP-C extreme-pressure lubricity tester. The extreme-pressure lubricity coefficient was obtained through this test. A lower lubricity coefficient indicates better lubricating performance of OAG. The calculation method for the lubricity coefficient and reduction rate is described as follows in Equations (4) and (5) [43]:

(4) $$K_{f}=\frac{34}{K_{w}}\times K_{d}/100$$

where K_f_ is the lubricity coefficient, K_w_ is the torque reading for deionized water, K_d_ is the torque reading corresponding to the drilling fluid during testing, and 34 is the correction factor.

(5) $$\Delta K_{f}=\frac{\left(K_{f}-K_{f1}\right)}{K_{f}}\times 100\%$$

where K_f_ is the lubricity coefficient before and after adding lubricant to the drilling fluid system or base slurry, and ΔK_f_ is the lubricity coefficient reduction rate.

#### 4.4.4. Adhesive Coefficient Test

The mud cake adhesion coefficient is used to characterize the lubricity of drilling fluid mud cakes and the static friction between the mud cake, the drill collar, and the wellbore wall. The mud cake adhesion coefficient of the water-based drilling fluid system or base slurry after adding OAG was tested using an NF-2 mud cake adhesion coefficient tester, and the mud cake adhesion coefficient was obtained. A lower adhesion coefficient indicates a better lubrication effect of OAG [44].

#### 4.4.5. Filtration Loss Test

Drilling fluid filtration loss testing includes API filtrate loss and high-temperature, high-pressure filtrate loss. The process for API filtration loss testing involves pouring the drilling fluid into a test cup, laying a filter paper flat, pressing it down, placing it into the instrument, applying a pressure of 0.69 MPa, and timing for 30 min. For high-temperature, high-pressure filtration loss testing, the drilling fluid is poured into a test cup, the upper and lower valves are closed, and then it is placed into a heating sleeve. The temperature is raised to the testing temperature, the pressure is adjusted to 3.5 MPa, and when a certain pressure is reached, the bottom valve is opened to start collecting filtrate for 30 min [45,46].

## Figures and Tables

**Table 1:** Lubrication coefficient test results of OAG on base slurry at different extreme-pressure times.

OAG/%	Lubrication Coefficient K_f_ at Different Time (Min)					Slider Heating Conditions
	5	10	15	20	30	
0	0.5412	0.5527	0.5619	0.5723	0.6074	heating
1.5	0.0324	0.0325	0.0331	0.0334	0.0328	unheating

**Table 2:** The effect of lubricant OAG concentration on the performance of base slurry and high-density drilling fluid under high-temperature and high-salt conditions.

Systems	Concentration of OAG/%	Conditions	*AV*/ mPa·s	*PV*/ mPa·s	*YP*/ Pa	*FL*_API_/ mL ^a^	*FL*_HTHP_/ mL ^a^	K_f_	ΔK_f_/ %
4% Freshwater-based slurry	0	AHR	10.5	6.5	4.0	24.7	- ^b^	0.874	- ^b^
	1.0	AHR	11.0	7.0	3.5	22.3	- ^b^	0.075	91.4
	1.5	AHR	11.0	7.5	3.5	21.6	- ^b^	0.037	95.8
High-density drilling fluid	0	BHR	86.5	65.0	21.5	1.9	12.5	0.247	- ^b^
		AHR	64.5	50.5	14.0	2.8	17.9	0.216	- ^b^
	1.0	BHR	90.5	68.5	22.0	1.4	11.8	0.095	61.5
		AHR	67.0	54.5	12.5	2.3	15.3	0.078	63.9
	1.5	BHR	92.0	69.5	22.5	1.2	11.5	0.081	67.2
		AHR	68.5	56.0	12.5	2.0	14.1	0.067	69.0

^a^ Filtration volume at API and HTHP conditions, respectively. ^b^ untested.

## Data Availability

The data presented in this study are openly available in article.
